# Stem Cell Based Gene Therapy in Prostate Cancer

**DOI:** 10.1155/2014/549136

**Published:** 2014-07-10

**Authors:** Jae Heon Kim, Hong Jun Lee, Yun Seob Song

**Affiliations:** ^1^Department of Urology, Soonchunhyang University, College of Medicine, Soonchunyang University Hospital, Seoul 140-743, Republic of Korea; ^2^Medical Research Institute, Chung-Ang School of Medicine, Seoul 156-756, Republic of Korea

## Abstract

Current prostate cancer treatment, especially hormone refractory cancer, may create profound iatrogenic outcomes because of the adverse effects of cytotoxic agents. Suicide gene therapy has been investigated for the substitute modality for current chemotherapy because it enables the treatment targeting the cancer cells. However the classic suicide gene therapy has several profound side effects, including immune-compromised due to viral vector. Recently, stem cells have been regarded as a new upgraded cellular vehicle or vector because of its homing effects. Suicide gene therapy using genetically engineered mesenchymal stem cells or neural stem cells has the advantage of being safe, because prodrug administration not only eliminates tumor cells but consequently kills the more resistant therapeutic stem cells as well. The attractiveness of prodrug cancer gene therapy by stem cells targeted to tumors lies in activating the prodrug directly within the tumor mass, thus avoiding systemic toxicity. Therapeutic achievements using stem cells in prostate cancer include the cytosine deaminase/5-fluorocytosine prodrug system, herpes simplex virus thymidine kinase/ganciclovir, carboxyl esterase/CPT11, and interferon-beta. The aim of this study is to review the stem cell therapy in prostate cancer including its proven mechanisms and also limitations.

## 1. Introduction

The introduction of stem cells (SCs) in cancer gene therapy is attributed mainly to the powerful advantage of it being a vector or cellular vehicle. Stem cell based cancer gene therapy is based on its tumortropic property. The tumor homing ability of SCs holds therapeutic advantages compared to other vehicles such as proteins, antibodies, nanoparticles, and viruses. Viruses or nonmigratory vector-producing cells have been utilized but demonstrated many shortcomings in effective delivery of the therapeutic agents. Virus-mediated gene therapies have limitation because of the difficulty in tracking cancer cells [1].

Another uprising evidence for using stem cells in cancer therapy is that it is nowadays broadly accepted that cancer is a stem cell disease [2, 3].

Compared with conventional chemotherapy, suicide gene therapy using SCs had no significant adverse effect on the weight gain of the animals. Such safety and efficacy imply a great potential of SC cell line expressing transgenes of interest for biologic study or clinical application [2, 3].

Although there have been large studies, including lung cancer, colon cancer, melanoma, glioma and brain tumors, stem cell based gene therapy in prostate cancer has been less focused on.

In this inspection, we provide an overview of the use of SC based gene therapy for prostate cancer. We identify SC sources and review several possible mechanisms of activity. We offer a summary of the current status of SC based gene therapy of prostate cancer with its limitation and also identify opportunities for further probe. This review contains only preclinical studies because SC based gene therapy for translational clinical therapies is not used until now.

## 2. Stem Cell Sources

To date, in prostate cancer gene therapy, only mesenchymal stem cell (MSC) and neural stem cell (NSC) were used in preclinical studies.

### 2.1. Mesenchymal Stem Cells (MSCs)

MSCs are an attractive vector for activating the prodrug because they could transport the therapeutic drug directly within the tumor mass; moreover, systemic toxicity could be avoided.

One of the most important advantages of MSCs compared to other types of SCs is its feasibility of acquisition. MSCs can be isolated as a fraction of bone marrow cells or other adult tissues. They possess an extensive proliferative potential and the capacity to differentiate into various cell types [4].

MSC has advantages in its feasibility of isolation and expansions in culture. Isolation of MSCs could be achieved from almost every type of tissue, including bone marrow, adipose tissue, muscles, liver, dental pulp, placenta, amniotic fluid, and menstrual blood, or umbilical cord blood [5, 6].

MSCs from bone marrow (BM-MSCs) reside in small numbers (approximately 10 cells per million of mononuclear cells) but could be well expanded in culture due to their plastic ability.

Adipose tissue for MSCs is about 10 times more abundant compared to bone marrow. Adipose tissue-derived mesenchymal stem cells (AT-MSCs) possess similar properties with BM-MSCs; therefore, BM-MSCs and AT-MSCs are used most frequently in stem cell studies or therapies. Griffin et al. have demonstrated the feasibility of utilizing human fetal bone marrow derived-MSCs for gene therapy [7].

The primary advantage of fetal BM-MSCs over adult BM-MSCs is the prolonged life span* in vitro*, which helps time-consuming enrichment of the MSCs transduction with constitutive expression of the desired transgene by single colony selection [7].

Another merit of MSCs is their immunosuppressive properties, which could be explained by the lacking of major histocompatibility complex (MHC-II) and showing minimal MHC-I expression [7–9]. Through this immunosuppressive property, allogeneic MScs may substitute autologous MSCs in delivering the therapeutic agent in targeted tumor therapy.

Clinical studies using MSCs revealed the prevention of graft-versus-host disease with its safety regardless of the variation of human leukocyte antigen [10].

These findings indicate the possibility of clinical use of premanufactured MSCs, which is conceived to reduce time constraints and increase the accessibility of MSCs in instances of emergency or inherited genetic disorders [2, 11].

### 2.2. Neural Stem Cells (NSCs)

Neural stem cells (NSCs) are self-renewing, multipotent cells that generate the main phenotypes of the nervous system and have tumor-tropic abilities. They have been exploited in preclinical as well as in clinical studies, especially in enzyme prodrug gene therapies. NSCs can be harvested from fetal, neonatal, or postnatal issues [12]. Since it is not feasible to obtain and isolate NSCs in sufficient numbers, immortalized neural progenitor cell lines instead of NSCs were prepared and used in several preclinical studies of prodrug cancer gene therapy [11, 13–15]. The well-characterized NSC line is HB1.F3, which was derived from fetal brain at 15 weeks of gestation and is known to be multipotent, migratory, and nontumorigenic.

Recently, FDA approved the second human NSC clinical trial in recurrent high-grade glioma [16]. Established on the evidence of intrinsic tumor's tropic capacity of NSCs, a genetically modified immortalized human NSC line is expected in its possibility of prostate cancer treatment.

### 2.3. Other Stem Cells

Endometrial regenerative cells (ERCs) are other optional SCs for utilization in cancer gene therapy, which are isolated from menstrual blood. They showed inhibition of intracranial glioma growth, but no preclinical trials have been reported for prostate cancer treatment. ERCs are a population of mesenchymal-like cells and are characterized by pluripotent differentiation capacity and production of unique growth factors [17].

## 3. Stem Cell Delivery as a Vector

Gene-directed enzyme prodrug therapy known as well as virus-directed enzyme prodrug therapy or suicide gene therapy did not demonstrate a satisfactory final result in clinical trials in the past. The primary cause for this failure was the missing tumor specificity of this approach. However, SC based prodrug cancer therapy is quite different from the classical prodrug gene cancer therapies. The main differences from earlier versions of cancer gene therapies are tumor homing able of MSCs,* in vitro* preparation of therapeutic SCs by plastic ability, and having prodrug converting genes integrated as DNA provirus in therapeutic SCs. Stable and effective production of prodrug converting enzymes under strong retroviral promoter is a great advantage of stem cell delivery as a vector.

## 4. Stem Cell Tracking

For CD system, in which the conversion of the 5-FC to 5-FU can be quantified with the nuclear magnetic resonance spectroscopy (NMRS) [18, 19]. The radiolabelled fluoronucleo side analogues of thymidine such as 1-(2-deoxy-2-fluoro-D-arabinofuranosyl)-5-iodouracil (FIAU) and radiolabelled 9-[4-fluoro-3-(hydroxymethyl)butyl]guanine (FHBG) can be used for HSV-tk substrates [20, 21].

It has been shown that MSCs can be easily labeled with super paramagnetic iron oxide (SPIO) nanoparticles [22]. MSCs carrying two kinds of nanoparticles [23] (polylactic acid NPs and LNCs) were shown to retain their viability, differentiation, and tumor homing capacities [22, 24]. Recently, Lee et al. demonstrated the usability of magnetic nanoparticles (MNPs) in the tracking of NSCs (HB1.F3 CD cells).

## 5. Mechanisms of Stem Cell Based Gene Therapy

Considering the issue that MSCs play dual roles in tumor genesis through potentiating tumor growth, enhancement of neovascularization, or differentiating into tumor stromal fibroblasts, suicide genes are of particular interest because they facilitate the development of MSCs in antitumor therapy [25–28].

The novel strategy of stem cell based gene therapy in prostate cancer includes silencing gene expression, expression of intracellular antibodies blocking cells' vital pathways, and transgenic expression of caspases and DNases. SC based gene therapy consists of two phases of treatment. In the first step, the gene for a foreign enzyme (bacterial, yeast or viral) is delivered and targeted to the tumor by transduction of SCs. In the second step, the enzymatic activity of the gene product converts less toxic prodrug to cytotoxic substance at the tumor lesion. Therefore, the active cytotoxic substances produced by enzymatic process within transduced MSCs effectively demolish the neighboring or surrounding tumor cells, which is called bystander effect.

In addition, the process of killing tumor and therapeutic stem cells can induce host immune responses mediated by natural killer (NK) cells and T-cells, which is called distant bystander effect [29–31].

The success of SC based the gene therapy depends on several factors, which are the catalytic activity of the enzyme encoded by suicide gene, a suitable combination of prodrug enzyme, the migration ability of the SC vector to target tumor cells, sufficient transgene expression, and the extent of bystander effect [2].

Recently, it was reported indeed that expression of the bifunctional suicide gene* CD::UPRT* increases radio sensitization and bystander effect of 5-FC in PC cells [32].

Using bifunctional suicide gene, researchers have previously shown, in* in vitro* and* in vivo* studies, that the bystander effect mediated by* CD::UPRT* gene directed enzyme prodrug therapy without a direct cell to cell contactor functional gap junctions [32–34].

### 5.1. Migration and Homing

Tumor sites are a microenvironment with hypoxia and inflammation, which release many cytokines that attract SCs. This tumor-tropic homing capability, together with inhibitory effect on tumor genesis, provides a strong foundation for using the systemic injected SCs for the treatment of distant tumors not easily accessible [35, 36]. With this strong tumor-tropic capability, SCs could be ideal cellular vehicles of antitumor agents.

Although it has been suggested that MSCs could be entrapped passively in highly vascularized tissues, most likely in the lungs, after systemically injected [37], tissue damage in lungs or other organs was not observed after systemic SCs treatment [37]. The migration/homing ability of MSCs has been known to be affected by passage number and high confluence [37–39].

### 5.2. Bystander Effect

To date, bystander effect is considered to be the potential treatment mechanism in SC therapy. Bystander effect is achieved by the therapeutic consequences of the transfected tumor cells. Although distant tumor cells are not directly transduced, those distant tumor cells could regress by distant paracrine effect. There are currently five mechanisms suggested for bystander effects: (a) release of soluble formulations, (b) passage through gap junctions, (c) passive transportation, (d) stimulation of local microenvironment, and (e) endocytosis of apoptotic vesicles [3]. Among the SC-based prodrug therapy, the CD/5-FU system showed stronger local bystander effect than the herpes simplex virus- (HSV-) thymidine kinase (tk)/ganciclovir (GCV). The 5-FU diffuses efficiently within the tumor cells and could overcome gap junctions that it passages without direct cell-to-cell contact. The GCV triphosphate showed different mechanisms for the bystander effect that the HSV-tk/GCV induces the local inflammation and devascularization, which probably enhance the vascular permeability and the formulation [3].

## 6. Stem Cell Therapy of Prodrug-Converting Enzymes

Prodrugs are nontoxic, inactive compounds delivered systemically and converted into biologically active cytotoxic agents only at the targeted tumor region. SC based gene therapy using prodrug-converting enzymes enables tracking and infiltrating into the tumor, which represents one of the expected gene therapy approaches [33, 40].

To date, three types of prodrug-converting enzymes have been introduced in prostate cancer (Table 1). First, cytosine deaminase (CD)/5-fluorocytosine (FC) enzyme prodrug therapy has a long history and also has been investigated largely in prostate cancer. The nontoxic 5-FC is converted to the toxic 5-FU in tumor region. The cellular enzymes process the formulation to three cytotoxic antimetabolites: (a) 5-FdUTP, (b) 5-FUTP, and (c) 5-FdUMP. The SC based gene therapy using CD suicide therapy showed cytotoxic effects which are based on three properties: (a) formation of (5-FU) RNA, (b) 5-FU DNA complexes, and (c) thymidylate synthase [3]. Bcl-2 charges for the downregulation of mitochondrial pathways [41].

The CD/5-FU system has been improved by including the gene uracil phosphoribosyltransferase (UPRT) [32, 42]. Finally, the combination of both systems (CD/5-FU and UPRT) delivered simultaneously has also been achieved. The effect of 5-FU on cell growth arrest and apoptosis has been attributed to the ability of its metabolites to induce the level and activity of the tumor suppressor p53 [43].

The basis for the CD/5-FC system is the ability of bacterial or yeast enzyme CD to convert nontoxic prodrug 5-FC into cytotoxic 5-fluorouracil (5-FU). Although bacterial or yeast enzyme could play the role of converting, yeast CD shows much higher efficacy in converting the 5-FC than the bacterial CD. It has been shown that yeast CD produces a 15-fold higher amount of 5-FU than bacterial CD [44].

The ability of yeast CD-expressing AT-MSCs (CDy-AT-MSC) to target tumor sites and micrometastases and to have a low immunogenic potential was reported [32, 33]. Increased efficiency of 5-FC to 5-FU conversion is achieved through bifunctional yeast fusion gene* CD::UPRT* [32]. The gene product of a bifunctional chimeric protein shows at least 100-fold higher activity than native yeast CD [45]. For NSCs, there are two preclinical studies dealing with this system [23, 46]. Lee et al. reported that human NSCs encoding CD (HB1.F3.CD) labeled with MNP showed the favorable treatment outcome. In their report, systemically transplanted HB1.F3.CD SCs migrated toward the tumor and, in combination with prodrug 5-FC, the volume of tumor was significantly reduced. These findings may contribute to the development of a new strategy of target chemotherapy against prostate cancer, including distant metastatic cancer [23].

Another reported prodrug system is HSVtk in combination with GCV. GCV is a nontoxic purine analogue and is phosphorylated by the enzyme HSVtk and further by endogenous kinases. Final phosphorylated substance, GCV-triphosphate, inhibits DNA synthesis and leads to cell death via apoptosis [47].

The HSV-tk/GCV system, induced a delay in the S phase and G2-phase. The observed apoptosis is not a direct result of the system activation, but a result of the delayed proliferation process. In addition, the induction of mitochondrial damage was observed [48], and caspase-8, Chk1 activation was associated with extensive cell death [49, 50].

Song et al. reported that the transduction of BM-MSCs expresses HSVtk using lent virus to inhibit the growth of subcutaneous PC3 prostate cancer xenografts as well as metastastic RIF-1 fibrosarcoma tumor in nude mice in the presence of GCV [51].

Lee et al. found out that the immortalization of human fetal bone marrow-derived mesenchymal stromal cells by simian virus 40 (SV40-hfBMSCs) could be a stable source of MSCs for clinical application of suicide gene therapy [39].

The CD/5-FC system is regarded more efficient than the HSVtk/GCV system for its stronger bystander effects [22]. The main reason is that 5-FU is a small molecule; therefore, it is able to enter neighboring cells through simple diffusion, while GCV requires gap junctions to affect surrounding cells.

Lastly, Yi et al. evaluated the tumor suppression effect of human NSCs genetically modified to express rabbit carboxyl esterase (rCE) enzyme (HB1.F3.CE), which can efficiently convert the prodrug CPT-11 into the cytotoxic drug SN-38 [46].

They have demonstrated that HB1.F3.CE cells increased the conversion rate of CPT-11 into SN-38, thereby inhibiting cancer cell growth more effectively. Therefore, the application of SC based CE gene therapy has the advantage of reducing the effective prodrug dosage. But this study has limitation because the setting of this study does not include* in vivo* study.

## 7. Stem Cell Therapy of Other Therapeutic Agents

Besides the prodrug therapy, there are also other options of SC gene therapies including other therapeutic agents such as interferon-*β* (IFN-*β*), interleukin (IL)-2, IL-7, and IL-12, and IL-18 [2]. Among these agents, genetic modification to overexpress interferon *β* was used in two preclinical trials (Table 1) [46, 52]. A significant increase in the natural kill cell activity was observed following IFN-*β* therapy correlating with the antitumor effect in prostate cancer model. Systemic level of IFN-*β* was not significantly elevated from this targeted cell therapy [52].

### 7.1. Limitations of Current Preclinical Studies and Future Perspectives

Although it has been well established that systemically administrated MSCs specifically migrate into tumor lesions, the role of recruited MSCs in the tumor microenvironment is still not fully elucidated yet. Several issues have to be clarified before consideration of clinical trials.

Although several preclinical studies showed favorable treatment outcome, the results were affected by transplanting SC amount. To date, appropriate dosages of SCs are not determined yet and it has to be elucidated before clinical trials. Results from preclinical studies suggested that the concentration of CD enzyme is crucial because it affects the reduction of tumor volume in proportion to the 5-FC dosage. Repeated delivery of CD-MSCs successfully suppressed the tumor growth [53].

The amount of SC vectors affects to the sensitivity of effect during suicide gene therapy [2, 33]. Thus, self-limiting the survival of SC vectors is also an important point, which is ideal for chemotherapy applications because the survival of delivered SCs needs to be no longer than sufficient duration for mediating the efficacy.

There is no guideline at the present time on the dosage of systemic injected MSCs as drugs in pharmaceutical sciences because the cellular kinetics of injected MScs is not well elucidated. The number and duration of MSC expressing suicide genes at the tumor sites would be an important determining factor for the therapeutic outcome because it poses the modulatory effects on tumor cells and neovascularization [26, 38, 54].

The different extent of the response to the cytotoxic effect of 5-FU is another issue to be clarified. Those responses observed between cancerous and noncancerous prostatic cells and between hormones sensitive and hormone-refractory PC cells are diverse due to the different enzyme expression in 5-FU metabolism. Indeed, Tanaka et al. have recently demonstrated that the expression of OPRT mRNA, which represents the capability to activate 5-FU, is significantly higher in PC tissue compared to normal prostate tissue [55].

The enzyme thymidylate synthase is also expressed at higher levels in the PC tissue than in the normal prostatic tissue. The possible reason is that the levels of OPRT activity increase in rapidly growing cells including tumor cells [56].

Transplanted SC effect on tumor genesis is also a crucial issue. The acceleration of tumor formation in preclinical studies was observed when MSCs were connected with cancer cells. MSC cells may support subcutaneous tumor growth when connected with tumor cells [57, 58]. The possible mechanisms for this issue include growth factors production, immunosuppressive character of MSCs, and formation of favorable microenvironment for cancer cells. However, other studies showed that MSCs may inhibit tumor growth in preclinical studies [59, 60], and possess antitumor genic effects on some specific tumor such as Kaposi's sarcoma [35].

This controversial effect of transplanted SC to tumor formation could be solved by SC based gene therapy. SC based gene therapy using the CD/5-FC system possesses the advantage to be safe because of suicide gene presence. Tumor cells were eliminated not only by the administration of 5-FC but also by therapeutic cells themselves [34].

*In vitro* experiments, cell viability of the noncancerous epithelial prostate cells, were only slightly affected by the presence of the SC vectors. This higher resistance is promising outcome that noncancerous epithelial prostate cells could be spared from the local bystander effect of 5-FU produced by the SC vectors during targeting primary prostate tumors.

CD/5-FC has an additional limitation that 5-FC has a high clearance rate due to its character of an antimycoticum drug. Hence, additional pharmacokinetics studies will be needed to overcome this point.

In all preclinical studies, a total regression of all tumors was not achieved when SC vectors were administered systemically. This is probably due to less number of therapeutic cells reaching the tumor with this delivery route [25] and to the fact that therapeutic cells are administered at a later time-point. Authors believe that appropriate multiple injections of SC vectors could bring more striking results in terms of growth arrest as well as an earlier beginning of the therapy.

Despite the feasible availability of human adult MSCs from various tissues, there is limitation to use autologous MSCs from patients with osteogenesis imperfect or tumors because of the genetic defeats. Allogeneic MSCs are an alternative, but immune-compatibility is always of concern. Also, the transduction of MSCs and subsequent sterility and identifying tests for tracking and survival of therapeutic SCs require high techniques and the reproducibility in other researchers could be low.

Another immunogenic issue to be solved is the origin of enzymes. The most frequently used enzymes are of nonmammalian origin and differ from any circulating endogenous enzymes in human. Studies are needed to clarify the requirements of nonmamalian enzymes to be expressed in such concentrations for achievement of sufficient conversion of a prodrug.

To promote SC based gene therapy in the treatment of PCs, several things have to be considered. One measure is the administration of adjuvant SC vectors, which increases the expression of gap junction proteins [61]. However, this approach does not increase the number of MSCs at the targeted lesions, and also the involvement of gap junction in SC based gene therapy is not well elucidated yet. Another measure is to induce further inflammation at the tumor sites to promote MSC migration and homing, which is clinically more relevant because current site-specific irradiation induces death of tumor cells and surrounding cells and inflammation and cytokine release [62]. Increasing the amount or frequency of chemotherapeutic agents is not an appropriate method because of possible systemic inflammation and cytotoxicity to the MSCs as well. However, the diverse classes of therapeutic agents lead to the interaction with SC based gene therapy in a synergistic manner by different underlying mechanisms, and other possible options include chemoembolization and tumor-specific chemotherapy.

MSCs were engineered to express the secreted tumor necrosis factor for apoptosis inducing ligand (TRAIL), which induce caspase-mediated apoptosis and could be applied to prostate cancer treatment. Several studies are now reporting the positive effect of this MSCs-TRAIL system in pancreatic cancer treatment [14, 24].

There are favorable results upcoming with the preclinical use of SCs to treat PC. SC based gene therapy, including enzyme prodrug therapy represents a more specific, less toxic and tailored approach to treating PC. Eradication of microscopic metastases that evade current detection techniques would be translated in a longer survival of PC patients with a better quality of life. Importantly, MSCs could be easily derived from the same patient.

## 8. Conclusions

The previous gene therapy showed unfavorable outcome due to the inability of vectors carrying the suicide gene to reach distant tumor cells and the inefficient spread of the vectors within the tumor. Therefore the SC based suicide gene therapy holds great potential for the possibility of clinical application due to the inherent and privileged migratory and tumortropic nature of MSCs or NSCs. SC based gene therapy is a promising treatment option in heterogeneous tumors like the PC. By further studies, better characterizing of this therapy is needed. It might offer a hope in the treatment of late-stage PC patients, but it may also be applied to prevent formation of metastases in patients with organ-confined PC.

## Figures and Tables

**Table 1:** Studies with stem cell-based gene therapy in prostate cancer.

Study	Stem cell	Animal	Cancer cell	Transplantation	Prodrug injection	*in vivo* tracking	Prodrug system	Injected dose of therapeutic stem cells	Transduction	Therapeutic stem cell
Lee et al., [23]	Human NSC	C57BL/6 mice	TRAMPC2	Left ventricle injection, 5 weeks after injection of cancer cells	Intraperitoneally, 500 *μ*g/kg/day in three rounds of 5 consecutive days with a break of 2 days	MNP	Bacterial CD/5-FC	1 × 10^6^ cells/100 *μ*L saline	Retrovirus	HB1.F3.CD

Song et al., [51]	Rat BM-MSC	Nude mice	PC3	Intravenous, 10 and 20 days after injection of cancer cells	Intramuscularly, 30 mg/kg each time for 5 consecutive days for 2 cycles,	GFP	HSV-tk/GCV	1 × 10^6^ cells/kg	Lentivirus	TK-BMSC

Cavarretta et al., [32]	Human AT-MSC	Nude mice	Du145, PC3 LNCaP PC3 (only for *in vivo*)	Intravenous, same time with cancer cell injection	Intraperitoneally, 500 *μ*g/kg/day daily, starting on second day after tumor appearance up to 27 or 32 days	None	yeast CD: UPRT/5-FC	2 × 10^6^ cells/200 *μ*L PBS	Retrovirus	CDy-AT-MSC

Ren et al., [52]	Mice BM-MSC	C57BL/6 mice	TRAMPC2	Intravenous, twice, 10 days after injection of cancer cells	None	GFP	None	5 × 10^5^ cells/200 *μ*L saline	Adenoassociated virus	MSC-AAV-IFN-*β*

Yi et al., [46]	Human NSC	None	LNCaP	None	None	None	Bacterial CD/5-FC, Rabbit CE/CPT-11, Bacterial CD/Human IFN-*β*	None	Leukemia virus	HB1.F3.CE, HB1.F3.CD, HB1.F3.CD.IFN-*β*

Lee et al., Immortalized [39]	Human BMSC	Nude mice	Du145, PC3	Intravenous, 10 days after injection of cancer cells	Intraperitoneally, 30 mg/kg in every round for 5 consecutive days	GFP	HSV-tk/GCV	1 × 10^6^ cells/kg	Lentivirus	SV40-TK-hfBMSC

NSC: neural stem cell; AT-MSC: adipose tissue derived mesenchymal stem cell; MNP: magnetic nanoparticles; BM-MSC: bone marrow derived mesenchymal stem cell; BMSC: bone marrow stromal cell; GFP: green fluorescent protein; UPRT: uracil phosphoribosyltransferase; CD: cytosine; CD/5-FC: cytosine deaminase/5-fluorocytosine; HSV-tk/GCV: herpes simplex virus-thymidine kinase/ganciclovir.
